# Development and Characterization of High-Throughput *Caenorhabditis elegans* – *Enterococcus faecium* Infection Model

**DOI:** 10.3389/fcimb.2021.667327

**Published:** 2021-04-29

**Authors:** Alexey V. Revtovich, Elissa Tjahjono, Kavindra V. Singh, Blake M. Hanson, Barbara E. Murray, Natalia V. Kirienko

**Affiliations:** ^1^ Department of BioSciences, Rice University, Houston, TX, United States; ^2^ Division of Infectious Diseases, McGovern Medical School, University of Texas Health Science Center, Houston, TX, United States; ^3^ Center for Infectious Diseases, School of Public Health, University of Texas Health Science Center, Houston, TX, United States; ^4^ Department of Microbiology and Molecular Genetics, McGovern Medical School, University of Texas Health Science Center, Houston, TX, United States

**Keywords:** *C. elegans*, *E. faecium*, high-throughput screen, virulence, host-pathogen interaction

## Abstract

The genus *Enterococcus* includes two Gram-positive pathogens of particular clinical relevance: *E. faecalis* and *E. faecium*. Infections with each of these pathogens are becoming more frequent, particularly in the case of hospital-acquired infections. Like most other bacterial species of clinical importance, antimicrobial resistance (and, specifically, multi-drug resistance) is an increasing threat, with both species considered to be of particular importance by the World Health Organization and the US Centers for Disease Control. The threat of antimicrobial resistance is exacerbated by the staggering difference in the speeds of development for the discovery and development of the antimicrobials versus resistance mechanisms. In the search for alternative strategies, modulation of host-pathogen interactions in general, and virulence inhibition in particular, have drawn substantial attention. Unfortunately, these approaches require a fairly comprehensive understanding of virulence determinants. This requirement is complicated by the fact that enterococcal infection models generally require vertebrates, making them slow, expensive, and ethically problematic, particularly when considering the thousands of animals that would be needed for the early stages of experimentation. To address this problem, we developed the first high-throughput *C. elegans–E. faecium* infection model involving host death. Importantly, this model recapitulates many key aspects of murine peritonitis models, including utilizing similar virulence determinants. Additionally, host death is independent of peroxide production, unlike other *E. faecium–C. elegans* virulence models, which allows the assessment of other virulence factors. Using this system, we analyzed a panel of lab strains with deletions of targeted virulence factors. Although removal of certain virulence factors (e.g., *Δfms15*) was sufficient to affect virulence, multiple deletions were generally required to affect pathogenesis, suggesting that host-pathogen interactions are multifactorial. These data were corroborated by genomic analysis of selected isolates with high and low levels of virulence. We anticipate that this platform will be useful for identifying new treatments for *E. faecium* infection.

## Introduction

Enterococci, particularly *Enterococcus faecalis* and *E. faecium*, are categorized as serious threats by the Centers for Disease Control and Prevention and since the 1970s have risen to be some of the most commonly isolated Gram-positive organisms responsible for nosocomial infections. In 2017 alone, there were approximately 55,000 infections with enterococcal species in the United states, resulting in 5,400 deaths (CDC, 2019). Infections commonly take the form of endocarditis, urinary tract infections (UTI), peritonitis, or meningitis, amongst others (Goh et al., 2017). Healthcare costs to treat enterococcal infection reached $540 million (CDC, 2019). Frequently, infections are acquired in healthcare settings, leading to endangerment of immunocompromised patients, including those who are infected with SARS-CoV-2 (Ramos-Martinez et al., 2020; Kampmeier et al., 2020; Bonazzetti et al., 2021). Enterococci are increasingly developing resistance to multiple antimicrobial drugs and disinfectants, including vancomycin, the drug of last resort (Kafil and Asgharzadeh, 2014; Levitus et al., 2020). In fact, about 30% of healthcare-associated enterococcal infections are resistant to vancomycin (CDC, 2019) and vancomycin-resistant enterococci (VRE) (the vast majority of which are *E. faecium*) often develop resistance to other antibiotics, leading to the requirement for intensive and invasive treatments. Resistance prolongs hospitalization and increases the likelihood for patients to contract secondary infections.

*E. faecalis* and *E. faecium*, both of which are commonly found as intestinal commensals (Huycke et al., 1998), are the two most commonly isolated enterococcal species in clinical samples. Although *E. faecalis* historically was better known as a human pathogen, *E. faecium* has risen to comprise over one-third of cases (Iwen et al., 1997; Treitman et al., 2005; Goh et al., 2017). This rise has led to suggestions that it may have recently acquired new virulence determinants (Kafil and Asgharzadeh, 2014; Davis et al., 2020) in addition to its frequent resistance to vancomycin, ampicillin, and other antimicrobials (Murray, 2000; Mundy et al., 2000).

Although it has been studied for several decades, relatively little is known about the molecular mechanisms involved in infection and pathogenesis in *E. faecium* (Freitas et al., 2018; Gao et al., 2018). Like most Gram-positive bacteria, it possesses a polysaccharide capsule and teichoic acid derivatives in its cell wall. It also includes several secreted factors, a secreted adhesion factor known as SagA, and a broad array of other attachment factors, including the *ebpABC_fm_* pilus operon (Hendrickx et al., 2008; Sillanpää et al., 2010; Montealegre et al., 2016), collagen adhesins (*acm*, *scm*) (Nallapareddy et al., 2006; Nallapareddy et al., 2008; Sillanpää et al., 2008), and surface proteins (*fms*, *esp*) (Willems et al., 2001; Hendrickx et al., 2007; Sillanpää et al., 2008). Most of these virulence factors have been demonstrated to contribute to *E. faecium* virulence in murine endocarditis and/or UTI models. However, it is quite likely that many other virulence determinants remain undiscovered.

Unfortunately, the model organisms available to study *E. faecium* virulence are predominantly mammals. Although effective, these models are strongly constrained in terms of throughput, are expensive, and are fraught with ethical concerns. This makes them inappropriate for large scale screens to identify novel virulence factor or therapeutic compounds (Garsin et al., 2014; Goh et al., 2017). In contrast, *Caenorhabditis elegans* infection models are cheap, fast, and share a surprising amount of infection biology with human beings. A *C. elegans* – *Enterococcus* pathosystem had previously been developed (Garsin et al., 2001), however only *E. faecalis* was able to cause persistent infection in *C. elegans* gut and kill adult worms. Unfortunately, although *C. elegans* has generally proven susceptible to infection with *E. faecalis*, it has been difficult to develop an infection system that is lethal, uses relevant pathogenic determinants, and is amenable to high-throughput techniques for *E. faecium*. For example, an agar-based system was developed that involved anaerobic growth of *E. faecium* (Moy et al., 2004), but killing depended upon hydrogen peroxide production. The prolific capacity of mammalian cells to produce catalase makes this virulence determinant less relevant in human infection. Another study indicated that *C. elegans* could be killed with *E. faecium* provided that one of several innate immune pathways were compromised (Yuen and Ausubel, 2018), but the virulence determinants involved were not identified and using worms with signaling defects may result in spurious outcomes.

In this study, we developed a liquid-based *C. elegans-E. faecium* pathogenesis assay. This assay was based on our conventional *P. aeruginosa* Liquid Killing assay (Kirienko et al., 2013; Anderson et al., 2018), which was modified for *Enterococcus* growth and to automatically quantify death, enabling unbiased and high-throughput studies. Using this assay, we tested available *E. faecium* lab strains containing mutations in several known virulence factors. We found that, although *C. elegans* death was not correlated with bacterial growth, highly virulent *E. faecium* strains possessed greater host colonization ability. We also tested the pathogenesis of ~120 *E. faecium* isolates, originating from hospital, commensal, microbiota, and animals, and observed that none of the clades had dramatically increased or reduced virulence. This marks an important step towards understanding the pathogenicity of *E. faecium* and will aid in the search of new therapeutic procedures.

## Materials and Methods

### Strains

*C. elegans* strains SS104 [*glp-4*(*bn2*)], N2 wild type, and *pmk-1(km25)* were maintained on standard nematode growth medium (NGM) (Stiernagle, 2006) seeded with *Escherichia coli* strain OP50 as a food source at 15°C, unless otherwise noted (Stiernagle, 2006). *C. elegans – E. faecium* experiments were performed at 25°C to induce sterility of temperature-sensitive *bn2* allele, similarly to other *glp-4(bn2)*-based assays (Moy et al., 2009; Kirienko et al., 2014; Kim et al., 2020). For experiment with N2 and *pmk-1(km25)*, sterility was induced by feeding worms with *cdc-25.1(RNAi)*-expressing *E. coli*. Absence of progeny facilitates analysis of assay outcomes.

*E. faecium* strains used in this study were collected over the past 30 years. The goal of our study was to compare the virulence of *E. faecium* belonging to different clades in *C. elegans* model. Therefore the strains representing clinical strains (mostly clade A1), commensal strains from healthy volunteers (mostly clade B), and isolates from animal sources or published clade A2 strains were selected. These isolates included 75 health care-derived isolates (mostly clade A1) from USA and Colombia (Arias et al., 2009; Sillanpää et al., 2009; Tran et al., 2013; Diaz et al., 2014), 21 human commensal strains (mostly clade B) (Sillanpää et al., 2009), and 21 isolates derived from animals, animal feed or obtained from published clade A2 strains studies (Sillanpää et al., 2009; Lebreton et al., 2013). Source information for these isolates is provided in **Table S1**. Genotype of strains with deletions is in **Table 1** below.

**Table 1:** List of *E. faecium* mutants used for this study.

Strain	Description	Reference
**TX0016**	WT	(Qin et al., 2012)
**TX1330**	WT	(Coque et al., 1995)
TX6060	TX1330 *Δgls33-glsB::erm(B), EryR*	(Choudhury et al., 2011)
TX6067	TX1330 *Δgls33-glsB::erm Δgls20-glsB1, EryR*	(Choudhury et al., 2011)
**TX2518**	WT	(Kim et al., 2010)
TX6089	TX2158 *Δfms21-20*	This study
**TX2154**	E1162, WT	(Heikens et al., 2007)
TX6111	E1162 *acmY176A, F192A Δfms18 Δscm*	This study
TX6114	E1162 *Δfms15*	This study
TX6109	E1162 *Δscm*	This study
TX6110	E1162 *acm* ^Y176A,F192A^ *Δscm*	This study
**TX0082**	WT	(Nallapareddy et al., 2006)
TX6115	TX0082 *ΔwxlC*	(Galloway-Peña et al., 2015)
TX6094	TX0082 *acm* ^Y176A,F192A^ *Δfms11 Δscm*	This study
TX6108	TX0082 *ΔwlcB*	
TX6086	TX0082 *Δacm*	(Somarajan et al., 2014)
TX5645	TX0082 *ΔebpABCfms(1-5-9)*	(Heimer et al., 2010)
TX6127	TX0082 *ΔccpA*	(Somarajan et al., 2014)
TX6137	TX0082 *ΔwxlABC*	
TX6135	TX0082 *ΔwxlA*	
TX6097	TX0082 *Δfms11 Δscm*	This study

Wild-type strains are indicated in bold.

### *E. faecium* Gene Deletions

Gene deletion mutants of previously described cell wall anchored (CWA) surface proteins with Ig-like folds (Sillanpää et al., 2008) were given *fms* designations and were generated in two clinical *E. faecium* strains (TX0082 and TX2154) using previously published methods (Panesso et al., 2011; Somarajan et al., 2014). Deletions included *fms18* (*ecbA* of *E. faecium* microbial surface component recognizing adhesive matrix molecules (MSCRAMM) (Sillanpää et al., 2008; Hendrickx et al., 2009); *fms11* (predicted pilus-associated) (Sillanpää et al., 2008) and *scm* (second collagen adhesin of *E. faecium*) (Sillanpää et al., 2008). Two substitutions (Y176A and F192A) were introduced in *acm*, a previously described MSCRAMM gene (Nallapareddy et al., 2003; Nallapareddy et al., 2008) using published methods (Liu et al., 2007). These strains were designated as TX6111 (E1162 *acm* ^Y176A,F192A^ *Δfms18 Δscm*), TX6114 (E1162 *Δfms15*), TX6109 (E1162 *Δscm*), TX6110 (E1162 *acm* ^Y176A,F192A^ *Δscm*), TX6094 (TX0082 *acm* ^Y176A,F192A^ *Δfms11 Δscm*), and TX 6097 (TX0082 *Δfms11 Δscm*) and have been listed in strain list in **Table 1**.

### *C. elegans* - *Enterococcus* Pathogenesis Assays

For the high-throughput, liquid-based pathogenesis assay, 25 synchronized young adult worms were sorted into 384-well plate. *E. faecium* strains were grown in deep-well plates at 700 rpm for 16 h. S Basal medium was mixed with brain-heart infusion (BHI) medium (10%) and *Enterococcus faecalis* or *E. faecium* (final OD600: 0.03) were then added into each well. Plates were incubated at 25°C until assay completion. At various time points, bacterial OD was measured with a Cytation5 (BioTek Instruments) plate reader and then plates were washed three times. Sytox Orange nucleic acid stain was added into each well and incubated for 12 - 16 h to stain dead worms. Plates were then washed three times, worms were transferred into a new 384-well plate, washed three more times, and imaged with Cytation5 automated microscope. Dead worms were quantified with CellProfiler software, similar to previously established pipelines (Conery et al., 2014).

For treatment with catalase, catalase (7.5 mg/mL) was supplemented in S Basal, BHI, *Enterococcus* mixture that was then distributed into each well of the 384-well plates.

For killing on agar, 50 young adult worms were transferred onto *Enterococcus*-BHI plates and incubated at 25°C. Worms were scored every day for survival; dead worms were removed from assay plates.

### Murine Peritonitis Model

*E. faecium* strains, TX0016 (clade A) and a commensal isolate, TX1330 (clade B) were tested following our previously published method (Singh et al., 1998). In brief, mice were injected intraperitoneally with appropriate dilutions of bacteria grown in BHI broth, premixed with sterile rat fecal extract (SRFE) and were observed for 5 days for survival. Data (h) were used to compare animal survival/mortality using six mice per group. Comparison of the survival curves was performed using a log-rank test with GraphPad Prism 4 for Windows^®^. A *p* < 0.05 was considered significant. All experiments were approved by the Animal Welfare committee, University of Texas Health Science Center at Houston.

### Colony Forming Unit (CFU) Assay

75 worms from liquid-based assay were collected into microcentrifuge tubes. Worms were washed three times to remove residual bacteria. After the final wash excess media was aspirated down to 100 µL. Equal volume of carbide beads was then added into the tubes and vortexed vigorously for 1 min to break worms. Supernatant was serially diluted and plated on BHI plates.

### Genomic Analysis

Genomic analysis was performed in the usegalaxy.org platform. FASTQ files generated from NGS were submitted for SNPs analysis to *snippy* (Galaxy version 4.5.0), a bioinformatic tools for rapid bacterial variant calling. Meanwhile, FASTA files were processed with *prokka* (Galaxy version 1.14.5) for genome annotation, *roary* (Galaxy version 3.13.0) for genomic alignment, and *RAxML* (Galaxy version 1.0.0) to construct a maximum-likelihood phylogenetic tree. **Figure 8A** was generated with R packages treeio (version 1.14.3) and ggtree (version 2.4.1).

### Microscopy

Upon 48 h of infection with *E. faecium* E007 in the liquid-based assay, worms were washed three times to remove residual bacteria, incubated for one hour with or without 20 µg/mL gentamicin, washed three times, and stained with 40 µM acridine orange for 4 hours. Worms were then washed three more times to remove unbound dye. Worms were imaged using a Zeiss ApoTome.2 Imager.M2 fluorescent microscope (Carl Zeiss, Germany) with a 20x objective magnification.

### RNA Interference Protocol

RNAi-expressing *E. coli* HT115 were cultured and seeded onto NGM plates supplemented with 25 μg/mL carbenicillin and 1 mM IPTG. 8,000 synchronized L1 larvae were transferred onto RNAi plates and grown at 25°C for 64 hours prior to exposure to pathogens.

RNAi experiments in this study were conducted by using RNAi-competent HT115 obtained from the Ahringer RNAi library (Kamath et al., 2003) and were sequenced prior to use.

### Statistical Analysis

With the exception of the mouse infection model, all experiments were performed in at least three independent biological replicates. The number of worms used per biological replicates is listed in the corresponding legends.

Student’s *t*-test analysis was performed to calculate the *p*-values when comparing two groups in an experimental setting. *p-*values were indicated in graphs as follows: NS not significant, *p < 0.05, **p < 0.01, and ***p < 0.001.

**Figure 2** was generated with R package ggplot2 (version 3.3.3). Correlation coefficient and *p*-value between *C. elegans* survival and *E. faecium* OD600 were calculated and indicated on the figure.

**Figure 5** was generated with R package ggplot2 (version 3.3.3). K-means clustering was performed with the ‘kmeans’ function in R. *p-*values between Clade A1 and Clade B were indicated on graphs.

## Results

### *E. faecium* E007 Is Pathogenic to *C. elegans*

**Figure 1:**
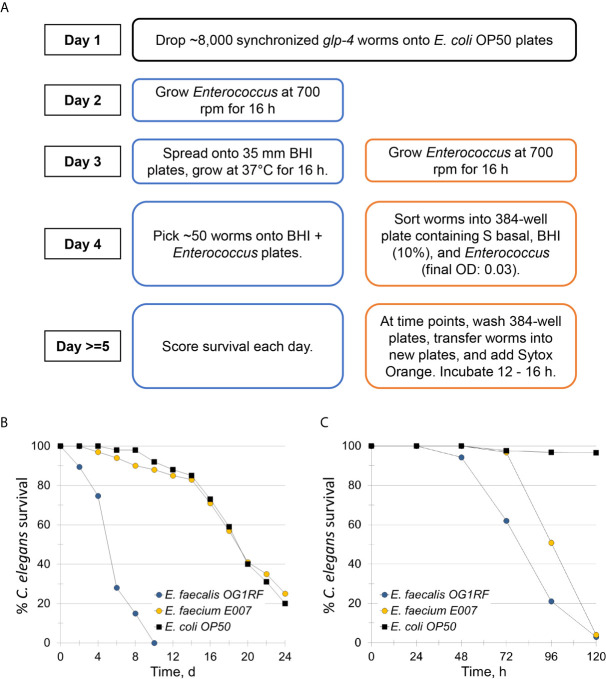
*E. faecium* E007 is pathogenic to *C. elegans* in the *Enterococcus* liquid-killing assay. **(A)** High-throughput, liquid-based *C. elegans* – *E. faecium* killing assay pipeline (right, compared to the conventional agar-based assay (left)). **(B, C)** Survival curves of *C. elegans* that were exposed to *E. faecalis* OG1RF, *E. faecium* E007, or non-pathogenic *E. coli* OP50 either in the **(B)** agar-based assay or **(C)** under liquid conditions. Three biological replicates were performed and analyzed; representative replicates are shown for **(B, C)**.

**Figure 2:**
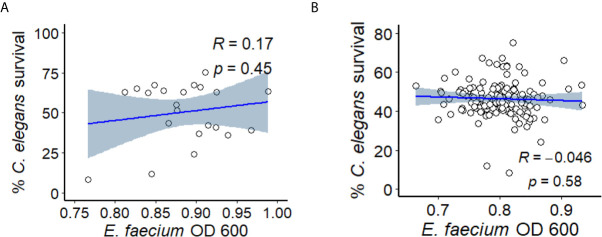
*E. faecium* virulence in *Enterococcus* liquid killing assay does not depend upon bacterial growth. **(A)** Correlation plot of a subset of ~30 *E. faecium* strains’ growth (OD 600, x-axis) against their virulence (based on *C. elegans*’ survival, y-axis). **(B)** Correlation plot of ~120 of *E. faecium* strains’ growth (OD 600, x-axis) against their virulence (based on *C. elegans*’ survival, y-axis). Three biological replicates with ~80 worms/replicate were analyzed. The correlation plot was generated with R package ggplot2 (version 3.3.3).

To study *E. faecium* virulence determinants, we developed the first high-throughput infection model for *E. faecium* that uses *C. elegans* as a host. In brief (see **Figure 1A**), synchronized, young adult *C. elegans* were sorted into 384-well plates. *E. faecium* or *E. faecalis* grown on BHI agar plates were then diluted into 10% BHI medium and added to worms at predetermined concentrations. Worms were infected at 25°C until assay completion and then were washed several times before staining with a cell-impermeant stain to quantify death. The entire assay can be performed within ~ 1 week, and ~90 different strains can be tested in parallel on a single 384-well plate. Unlike in previously published agar-based systems where only *E. faecalis* killed the worms (Garsin et al., 2001), our liquid assay showed killing within a week by both *E. faecalis* and *E. faecium* (**Figures 1B, C**). Importantly, we saw no significant correlation between bacterial growth (as determined by OD) and host death either when we used a pilot set of ~30 isolates or with the full set of ~120 isolates (**Figure 2**). This suggests that host death is a specific phenomenon related to an interaction between the host and the pathogen, and does not merely result from bacterial growth.

As noted above, *E. faecalis*-mediated killing of *C. elegans* in the previously described assay depended on hydrogen peroxide production (Moy et al., 2004). Although our assay does not involve anaerobic growth (which was required for hydrogen peroxide production), we tested whether the addition of catalase to our assay would influence rates of host death. In general, we saw no decrease in killing after the addition of catalase, consistent with catalase-independent pathogenesis (**Figure S1**).

### PMK-1/p38 MAPK, but Not MDT-15/MED15, Is Dispensable for *C. elegans* Resistance to Liquid-Based *E. faecalis* Pathogenesis

**Figure 3:**
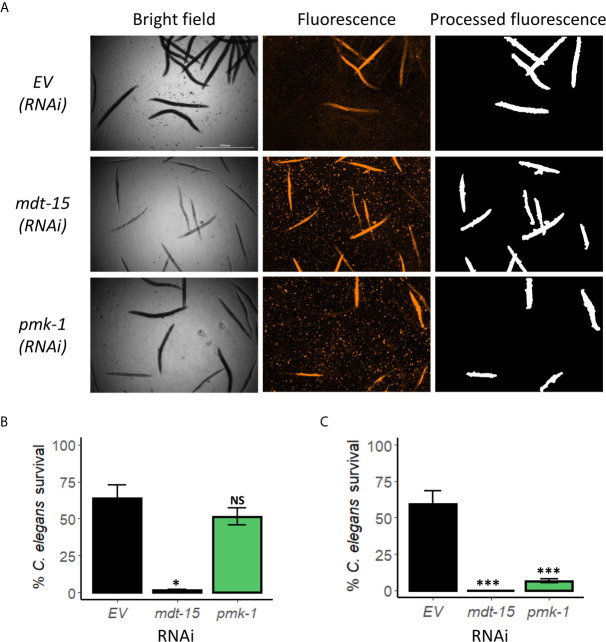
PMK-1 did not confer resistance towards *E. faecium* in *Enterococcus* liquid-killing assay. **(A)** Fluorescence images and **(B)** quantification of *C. elegans* survival after exposure to *E. faecium* E007 in liquid. Worms were reared on *E. coli* expressing empty vector (*EV*) as control or *mdt-15(RNAi)* or *pmk-1(RNAi)*. **(C)** Quantification of *C. elegans* survival at 120 h exposure to *E. faecium* E007 in agar. Three biological replicates with ~400 worms/replicate for liquid-based assay or ~180 worms/replicate for agar-based assay were analyzed. Error bars represent SEM. *p* values were determined from Student’s *t*-test. NS not significant, *p < 0.05, ***p < 0.001.

The PMK-1/p38 MAPK pathway is arguably the most important innate immune pathway for responding to bacterial infection in *C. elegans* and has been implicated in resistance in most agar-based pathogenesis assays (Kim et al., 2002; Bolz et al., 2010; Shivers et al., 2010; Pukkila-Worley et al., 2012). In addition, *pmk-1* mutation has been shown to increase sensitivity to *E. faecium* (Yuen and Ausubel, 2018). In contrast, the PMK-1/MAPK pathway is detrimental for survival in a liquid-based *C. elegans-P. aeruginosa* pathogenesis assay (Kirienko et al., 2015; Tjahjono and Kirienko, 2017). We used RNAi to knock down *pmk-1* and then tested young adult worms in the liquid *E. faecium* assay for increased host death. As with *P. aeruginosa*, loss of PMK-1 function did not increase *E. faecium*-mediated killing in the liquid-based assay (**Figures 3A, B** **)**. In contrast, disrupting PMK-1 function in the agar-based assay reduced survival (**Figure 3C**, **S2A**). These results were confirmed using a known loss-of-function mutation, *pmk-1(km25)* (**Figure S2B**).

Recently, we showed that the Mediator subunit MDT-15/MED15 has a role in host resistance against *E. faecalis* (Hummell et al., 2021). To probe whether this function is retained in this liquid-based assay, we also used RNAi to knock down *mdt-15* expression. Unlike *pmk-1(RNAi)*, *mdt-15(RNAi)* significantly increased host mortality in both liquid- and agar-based assays (**Figure 3**, **S2A**), suggesting that MDT-15 serves an innate immune function in these assays as well.

### *E. faecium* Strains From All Clades Showed Wide Range of Virulence

**Figure 4:**
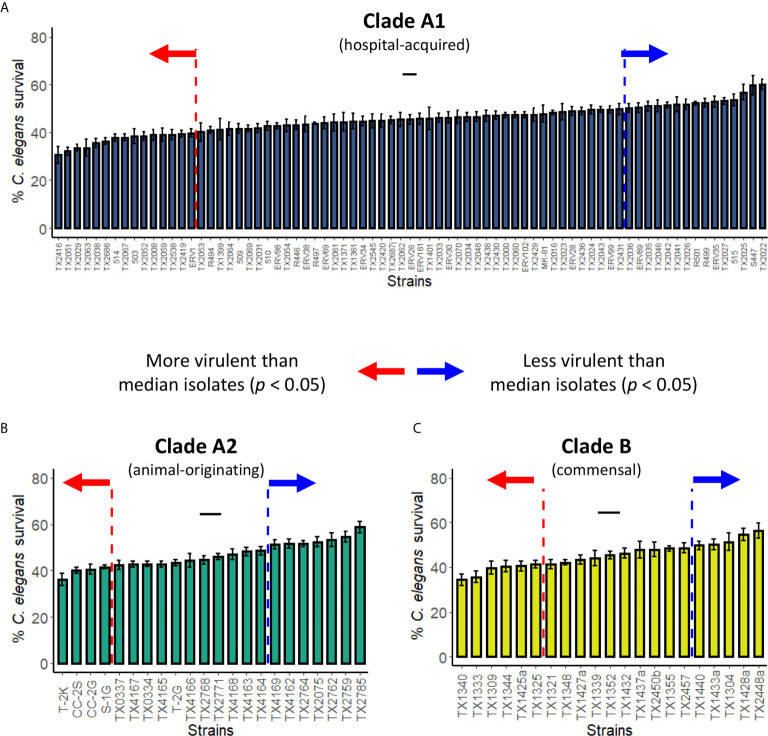
Pathogenicity distribution of *E. faecium* strains belonging to Clade A1, A2, or B *C. elegans* were exposed to *E. faecium* strains belonging to **(A)** Clade A1, **(B)** Clade A2, or **(C)** Clade B in the liquid-killing assay. At least three biological replicates with ~80 worms/replicate were analyzed. Error bars represent SEM. *p* values were determined from Student’s *t*-test against the median isolates (indicated with short black lines in the graphs). Arrows indicate all isolates that are more (red) or less (blue) virulent than the median isolates (*p* < 0.05).

Genome analysis of *E. faecium* strains indicates that they belong to at least two different phylogenetic clades: A (comprised of hospital-acquired strains and strains mostly derived from animal origins), and B (mostly human commensal strains) (Palmer et al., 2012; Galloway-Peña et al., 2012; Lebreton et al., 2013). Although earlier phylogenetic evidence suggests that clade A may be broken into two subgroups (Palmer et al., 2012; Galloway-Peña et al., 2012; Lebreton et al., 2013), a more recent, larger analysis called this conclusion into question (Raven et al., 2016). Pathogenicity of isolates from clade A1 (**Figure 4A**), clade A2 (**Figure 4B**), and clade B (**Figure 4C**) varied, ranging from 45-70% host mortality. Although we tested a larger number of strains from clade A1, all three clades showed similar patterns of variability in virulence. We determined median mortality for each clade and identified strains that were significantly more or less pathogenic for further analysis.

**Figure 5:**
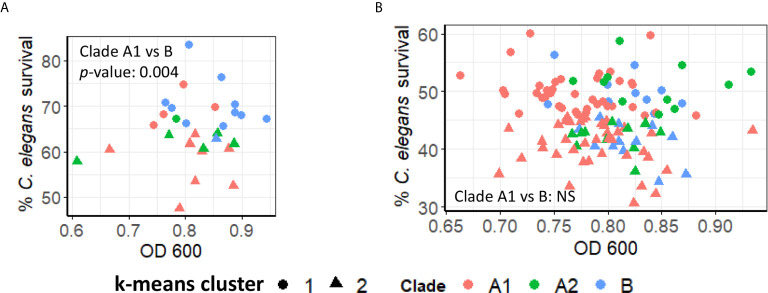
Pathogenicity of *E. faecium* from distinct clades does not differ significantly. **(A, B)** Pathogenesis of **(A)** preliminary (~30) or **(B)** all available (~120) *E. faecium* strains belonging to Clade A1, A2, or B In **(A, B)**, *C. elegans* survival (y-axis) were plotted against *E. faecium* OD 600 (x-axis). Clustering was performed with ‘kmeans’ function (R v4.0.3). All panels show pooled results from three biological replicates. *p*-values were determined from Student’s *t*-test and are indicated on graphs.

An initial analysis of the pathogenicity of ~30 isolates in our liquid-based model was performed. K-means clustering was then used to group the strains based on virulence, resulting in strong preferential grouping of A1 and A2 clades in high virulence and B strains in low virulence (**Figure 5A**). However, a more robust analysis, using a total of ~100 strains, abolished this difference, with each clade being approximately equally represented in the high- and low-virulence groups (**Figure 5B**).

### Strains With Higher Virulence Had Higher Colonization Ability in the Liquid-Based Assay

**Figure 6:**
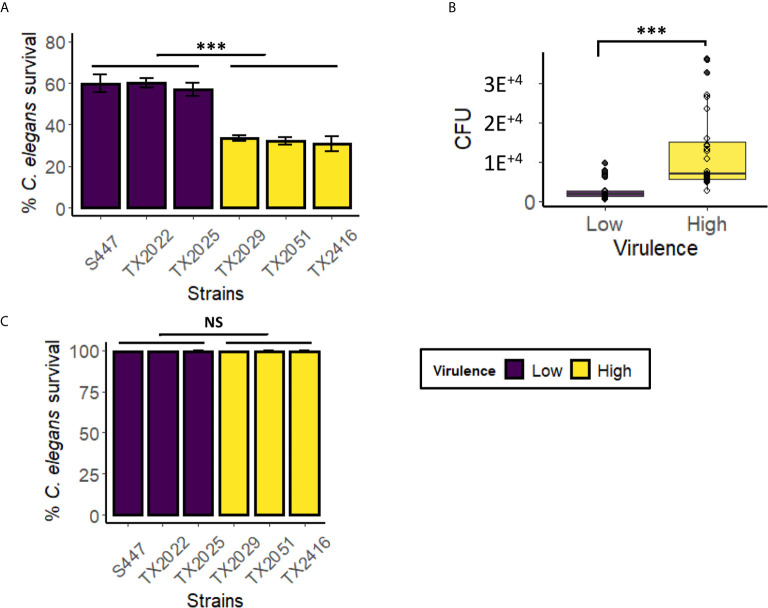
Highly virulent *E. faecium* strains showed higher colonization, compared to low virulence strains, in the *Enterococcus* liquid-killing assay. Six *E. faecium* strains (three with the highest virulence in the liquid-based assay and three with the lowest virulence) were exposed to *C. elegans* in liquid-based assay. Panel **(A)** shows strain pathogenesis (*C. elegans* survival), while panel **(B)** shows pooled average of CFU from *C. elegans* gut after colonization. CFU counts for individual strains are in **Figure S3**. **(C)** The killing ability of bacteria-free filtrates (from isolates in panel A) was examined after 100 h incubation with *C. elegans*. Three biological replicates with ~60 worms/replicate for CFU assay or ~400 worms/replicate for pathogenesis assay were analyzed. Error bars represent SEM. *p*-values were determined from Student’s *t*-test between pooled low- and high virulence groups. NS not significant, ***p < 0.001.

Colonization is the first step in any infection, including bacterial infections of *C. elegans* with *E. faecalis* or *E. faecium* (Tan et al., 1999; Aballay et al., 2000; Garsin et al., 2001; Kurz et al., 2003; Goh et al., 2017). In contrast, our previously reported liquid-based *P. aeruginosa* assay, which is based on intoxication rather than infection, showed relatively few bacteria in the intestine, more consistent with transit of undigested bacteria, rather than intestinal colonization (Kirienko et al., 2013). However, in liquid-based *C. elegans* – *Candida albicans* model, host colonization was observed (Rosiana et al., 2021). To test whether colonization is related to virulence in *C. elegans- E*. *faecium* liquid assay, we selected six strains from Clade A1, including the three with the highest (TX2029, TX2051, and TX2416) and the lowest (S447, TX2022, and TX2025) virulence, and infected *C. elegans* with them in the liquid assay (**Figure 6A**). After 48 hours of infection, some of the worms were harvested, washed thoroughly to remove extracorporeal bacteria, and then physically disrupted to release intestinal bacteria. Lysates were serially diluted and plated on BHI media to determine bacterial titer *via* CFU counting, allowing colonization to be directly measured. We found that the high-virulence strains as a group had a better ability to colonize *C. elegans* than low-virulence strains (**Figure 6B** and **Figure S3**, *p* < 0.001, pooled low- and high- virulence groups). To ensure that only bacteria from the worm alimentary track, but not those that adhere to the cuticle, contributed to CFU counts, we performed the same assay except that worms were treated with gentamicin for 1 h after the first three washes (**Figure S4**). Worms were then incubated with 40 µM acridine orange, a dye commonly used to stain bacteria (Neeraja et al., 2017). Fluorescent imaging confirmed that bacteria were only detected in the worms’ gastrointestinal tract, both for gentamicin-treated and untreated sub-populations, but not on the cuticles (**Figures S4A, B**). These results suggested that the ability to persist in *C. elegans* gut may be an important contributor to *E. faecium* virulence in this liquid assay.

In a previously-published liquid-based *C. elegans* pathosystem, we showed that toxin secretion, particularly of the siderophore pyoverdine, was very important for host killing by *P. aeruginosa* (Kirienko et al., 2015; Kang et al., 2018). In this case, spent media or purified pyoverdine were capable of killing worms even in the absence of live bacteria (**Figure S5**). To test the ability of secreted factors to affect host survival during *E. faecium* infection, the same panel of six *E. faecium* isolates was grown under assay conditions, but in the absence of worms, for four days. Then bacteria-free filtrates were produced and *C. elegans* were exposed to them for ~100 h (**Figure 6C**). Virtually no host death was observed, suggesting that live bacteria and host-pathogen interactions are the driving force of pathogenesis in this assay.

### Some *fms* genes Are Important for Pathogenesis in the *C. elegans – E. faecium* Liquid Killing Assay

*E. faecium* infection is a multifactorial process that includes attachment, colonization, and biofilm formation. To determine which virulence factors are important for killing in liquid, we measured the pathogenicity of available *E. faecium* strains harboring deletions of targeted virulence factors (**Table 1**), including genes from the *gls* (Choudhury et al., 2011), *fms* (Sillanpää et al., 2008; Sillanpää et al., 2009; Sillanpää et al., 2010), *wxl* (Galloway-Peña et al., 2015), and *ccpA* (Somarajan et al., 2014) genes and gene families.

Deletion of genes encoding general stress proteins (Gls) *gls33-glsB* alone or doubled with *gls20-glsB1* did not appear to affect host survival (**Figure S6A**). This also seemed to be the case with mutants harboring deletions of genes in the WxL loci (Galloway-Peña et al., 2015). The WxL loci, are a series of three loci that each encode between 3 and 6 genes, including peptidoglycan-binding proteins (Brinster et al., 2007). Three strains with a single mutation each and one strain with triple mutations of the WxL loci did not significantly alter worms’ survival as compared to the parental strain TX0082 (**Figure S6B**).

**Figure 7:**
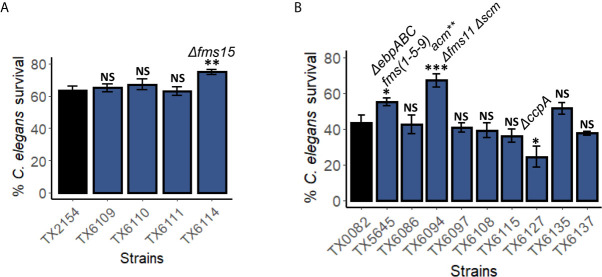
Genes encoding surface proteins and pili are important for pathogenesis in the *C. elegans – E. faecium* liquid-killing assay. Pathogenicity of several strains harboring mutations in **(A)** *fms10 (scm)*, *acm*, *fms18*, or *fms15*, or **(B)** *fms*, *scm*, *wxl*, or *ccpA* were assayed in the *C. elegans – E. faecium* liquid-based pathosystem. Three biological replicates with ~400 worms/replicate were analyzed. Error bars represent SEM. *p* values were determined from Student’s *t*-test. NS not significant, *p < 0.05, ** *p* < 0.01, ***p < 0.001.

On the other hand, the *E. faecium* surface proteins (Fms) of the MSCRAMM (microbial surface component recognizing adhesive matrix molecules) family were more likely to be involved in pathogenesis in this *E. faecium – C. elegans* killing assay. *fms* genes are tightly associated with *E. faecium* clinical isolates (Sillanpää et al., 2008). We tested multiple mutants with one (*scm*, *acm*, or *fms15*), two (*Δfms21-20*, *acm* ^Y176A,F192A^ *Δscm*, and *Δfms11 Δscm*), or three genes mutated (*acm* ^Y176A,F192A^ *Δfms18 Δscm*, *ΔebpABCfm*, and *acm* ^Y176A,F192A^ *Δfms11 Δscm*). Among the strains tested, the single deletion of *fms15*, an adhesin gene, significantly reduced *E. faecium* virulence (**Figure 7A**). Moreover, triple mutation of *acm* ^Y176A,F192A^ *Δfms11 Δscm* and deletion of pilus subunit proteins encoded by the three-gene locus *ebpABCfm* (*fms1-5-9*) reduced pathogenicity as well (**Figure 7B**). As the MSCRAMMs play a role in host-pathogen adherence, these results corroborated our finding that colonization and persistence in *C. elegans* gut is important for virulence.

Interestingly, deletion of *ccpA*, a regulator of carbon catabolite repression that affects *E. faecium* growth and is important for virulence (Somarajan et al., 2014), increased pathogenicity (**Figure 7B**). This result was unexpected as *ccpA* deletion mutant showed reduced biofilm formation, growth, and attenuated infection in endocarditis model (Somarajan et al., 2014).

### Genome Analysis of Clade A1 Strains

**Figure 8:**
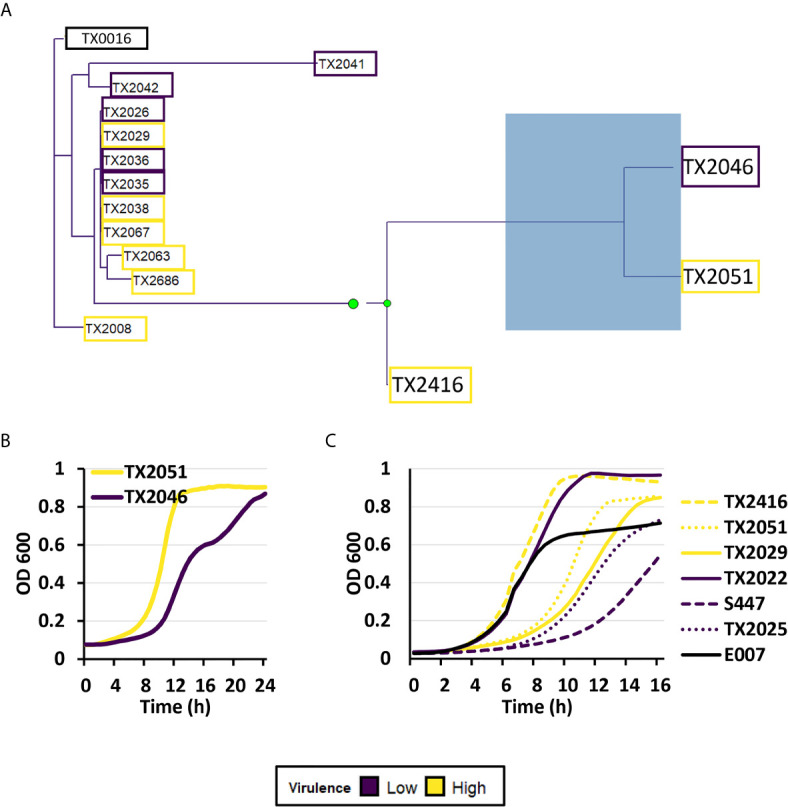
Genomic analysis showed a relationship between high- and low virulence strains. **(A)** A maximum-likelihood phylogenetic tree of eight strains belonging to the highest virulence group (yellow), six strains belonging to the lowest virulence group (purple), and a reference strain (TX0016, black). The phylogenetic tree was created by performing roary (Galaxy version 3.13.0), followed by RAxML (Galaxy version 1.0.0), and visualized by using treeio (v1.14.3) and ggtree (v2.4.1) packages (RStudio, R version 4.0.3). **(B)** Kinetic growth measurement or optical densities (OD 600) of TX2046 and TX2051 in BHI media. **(C)** Kinetic growth measurement or optical densities (OD 600) of three high- (TX2416, TX2051, TX2029) and three low-virulence isolates (TX2022, S447, TX2025), and reference strain E007 in BHI media. For **(B, C)**, three biological replicates were performed and analyzed; a representative replicate is shown.

To further understand *E. faecium* virulence mechanisms, we obtained whole genome sequencing data for eight strains in the higher virulence and six strains in the lower virulence groups. Strains from Clade A1 were selected for this analysis as they are most commonly associated with human infection. Phylogenetic tree construction showed close relationships among the fourteen strains, except for TX2046 (lower virulence) and TX2051 (higher virulence) (**Figure 8A**).

Genomic analysis showed that TX2046 contained two frameshift mutations: a one nucleotide insertion in *sorB* (a PTS system sorbose-specific EIIB component) and another in *mazE* (the gene encoding the MazE Type II antitoxin). TX2051 acquired a frameshift in *araQ* (*L*-arabinose transport system permease protein) and a stop codon in *wecA* (UDP-*N*-acetylgalactosamine-undecaprenyl-phosphate *N*-acetylgalactosamine phosphotransferase).

Toxin-antitoxin pairs are commonly found in bacterial pathogens, and mutation of the antitoxin *mazE* would result in greatly diminished bacterial survival. We performed growth analysis of the OD 600 for the two strains TX2046 and TX2051. We observed differences in growth rates, with TX2051 growing faster than TX2046 (**Figure 8B**). Thus, TX2051 might be more virulent due to faster growth and colonization rate. This result also showed that, at least in selected cases, pathogenicity may depend on bacterial growth. However, these two parameters did not correlate in a larger group (**Figure 8B** and **Figure S3**).

### The Liquid-Based *E. faecium* Model Recapitulates Virulence Observed in a Murine Peritonitis Model

**Figure 9:**
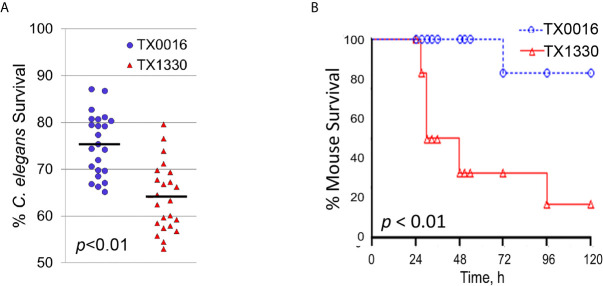
The *C. elegans – E. faecium* pathogenesis model recapitulates *E. faecium* virulence. **(A, B)** Survival curves of **(A)** *C. elegans* or **(B)** mice that were exposed to two *E. faecium* strains TX0016 and TX1330. All panels show results from pooled biological replicates (each dot in **A** represents a group of 20 worms). *p*-values were determined from Student’s *t*-test and are indicated on graphs.

Most importantly, our *C. elegans – E. faecium* pathogenesis model recapitulated virulence in a murine peritonitis model. Two strains that differ in origin, TX0016 [or DO, an endocarditis isolate with one of the first *E. faecium* genomes sequenced (Qin et al., 2012)] and TX1330 [a community-derived isolate (Qin et al., 2012)] were used to infect *C. elegans* or mice. Pathogenesis was noticeably reduced in TX0016 strain compared to TX1330 in both *C. elegans* (**Figure 9A**) and mice (**Figure 9B**).

## Discussion

In this study, we developed a *C. elegans* – *E. faecium* liquid-based pathogenesis assay that includes a lethal infection. Previous tests of *E. faecium*-infected *C. elegans* showed that the bacterium could effectively colonize the intestine of *C. elegans*, and even trigger an immune response (Garsin et al., 2001; Yuen and Ausubel, 2018), but the worms retained nearly normal life spans. Disrupting host immune effectors caused worms to succumb, indicating that this defense is productive. Interestingly, the death that we observed suggests that either a different pathogenic mechanism is used in this assay or that the normal defense mechanism has been compromised. This conclusion is bolstered by our observation that PMK-1/p38 MAPK innate immune pathway activity is not involved in survival.

This *E. faecium* screen was developed from our previously published *C. elegans – P. aeruginosa* liquid-killing assay (Kirienko et al., 2013) and median survival was typically 4 days post infection. This model allows high-throughput screens to be conducted for studying *E. faecium* virulence, an advantage other current model systems do not offer (Garsin et al., 2014; Goh et al., 2017). This enables identification of virulence factors by screening for increased host survival compared to wild-type bacteria.

Most importantly, our *C. elegans* model recapitulated features of virulence observed in a murine—*E. faecium* model. Despite their evolutionary distance, *C. elegans* retains many important features of the innate immune system (e.g., p38 MAPK, Nrf2, FOXO pathways), host responses (e.g., lysozymes, lectins, other effectors production), and metabolic adjustments seen in mammals (Irazoqui et al., 2010; Pukkila-Worley and Ausubel, 2012; Battino et al., 2018). Admittedly, we saw considerable differences in sensitivity in *C. elegans* vs mammalian models when using known attenuated mutants. However, the rapid generation time and low cost will allow this assay to be further refined to more accurately reflect mammalian infections. Meanwhile, this host-pathogen interaction model is capable of probing host and pathogen genetic factors that influence virulence. In addition, the small size of *C. elegans* allows this assay to be performed in 384-well plates, making it amenable for high-throughput screening and medicinal chemistry approaches.

Once initial studies with our screen are complete, a potential model system to validate the results of these screens is an organoid culture system. This culture system is currently thriving and many types, including intestinal and lung organoids, have been successfully established (Dutta and Clevers, 2017). Organoid systems can provide additional insights into the interplay between *E. faecium* and human bodies, particularly the inflammatory responses. The idea of finding therapeutics that may mitigate pathogenesis or stimulate hosts’ immune systems and potentially shift this pathogen back toward a commensal state is a tantalizing prospect. Since the rise and spread of resistance to treatment with these effects is likely to be slower than for conventional antimicrobials, this might also boost our ability to treat these infections moving forward.

Interestingly, we found no correlation between the initial bacterial titer of *E. faecium* and pathogenesis towards *C. elegans* in this system. Although some strains, like TX2051 and TX2046 (the latter is missing the MazE antitoxin, and shows slower bacterial growth), showed a correlation between faster growth and higher virulence, there was no overall trend. This is consistent with our observation that secreted virulence factors do not seem responsible and that colonization is required, as shown by diminished pathogenesis by the *fms15* mutant.

This finding, and the broader context of efficient intestinal colonization with *E. faecium* in this assay, was unexpected, given our previous results with a *C. elegans* – *P. aeruginosa* assay that has been utilized by our lab for a number of years. In the *P. aeruginosa* liquid pathogenesis assay, the living bacteria present in the intestine appear to be transient and have escaped the digestive process, but are not establishing a significant colonization. Consequently, isolates or mutants that would be attenuated on agar may have full virulence in liquid, and *vice versa*. In the case of *P. aeruginosa*, growth on agar (or in the host) leads to high bacterial density and key involvement of quorum-sensing pathways (Feinbaum et al., 2012). In liquid, growth is limited, quorum sensing has no role, and production of the siderophore pyoverdine drives a lethal host intoxication (Kirienko et al., 2015; Kang et al., 2018; Kang and Kirienko, 2020).

A recently published analysis of the role of *Candida albicans* adhesins in virulence using a liquid-based *C. elegans* model (Rosiana et al., 2021) reveals a third possibility. In this model, like the *P. aeruginosa* and *E. faecium* models, greater colonization is observed in an agar-based assay than a liquid assay, although colonization still occurs in liquid, like with *E. faecium*. Unlike either previous model however, pathogenesis and colonization are inversely correlated here. From comparing these systems, it is obvious that host-pathogen interactions are dynamic, depending upon virulence factors regulated by both participants, and that careful study is needed in each case.

Host defenses are also clearly context- and pathogen-dependent, and PMK-1/p38 MAPK can range from essential (*P. aeruginosa* infection on agar, *E. faecium* on agar) to dispensable (*E. faecium* infection in liquid) to detrimental (*P. aeruginosa* intoxication in liquid). Involvement of the Mediator complex subunit MDT-15/Med15 was recently shown to be required for increased survival of *C. elegans* exposed to both Gram-negative and Gram-positive bacteria (e.g., *P. aeruginosa* and *E. faecalis*) in liquid when treated with immune-stimulatory compounds (Hummell et al., 2021). Here *mdt-15*/Med15 knockdown resulted in dramatic susceptibility of *C. elegans* to *E. faecium* infection in liquid. This indicates that the involvement of Mediator complex into innate immunity may be a wide-spread phenomenon.

In this study, we tested ~120 *E. faecium* strains isolated from various clinical and environmental samples from many different geographical locations. Most of the strains belong to Clade A1, a group that includes members that are tightly associated with nosocomial infections and are notorious for their hypermutability, acquisition of assorted pathogenic determinants, and acquisition of resistance mechanisms (Lebreton et al., 2013; Freitas et al., 2018). Clade A and Clade B (commensal) have been estimated to have diverged approximately 3000 years ago (Lebreton et al., 2013; Raven et al., 2016), with the former carrying more genetic elements encoding ABC transporters for antibiotic transport and phosphotransferase systems for utilization of carbohydrates with a non-dietary origin. These features are predicted to make Clade A more likely to be resistant to antimicrobials and more pathogenic (Lebreton et al., 2013). To our surprise, however, each clade showed similar distribution of virulence, suggesting that each group has potential for threatening public health. Indeed, recent evidence showed that infection with Clade B isolates is increasingly frequent (Lebreton et al., 2018), much as was observed for *E. faecium* in general over the last 50 years. Troublingly, antibiotic resistance is spreading rapidly within populations in Clade B, and hybrid strains of Clade A1 and B are increasingly common (Galloway-Peña et al., 2012; Lebreton et al., 2013).

Recent studies profiling putative virulence markers in various *E. faecium* isolates with diverse origins agreed with this finding. Uneven distributions of several of these virulence markers were observed within the three clades (Qin et al., 2012; Freitas et al., 2018). While Clade A1 isolates carry more putative virulence markers than Clade A2 or B, most virulence markers are present in all three clades, including *acm*, *scm*, *fms15*, *fms21*, *ebpBC*, and WxL genes, indicating their importance for host colonization (Freitas et al., 2018). Meanwhile, *fms11*, *fms18*, *fms20*, and *ebpA* are mostly found within Clade A1. The presence of complete pili gene clusters, e.g., *ebpABC_fm_*, however, is tightly associated with ampicillin-resistant clinical isolates (Qin et al., 2012; Freitas et al., 2018).

We validated our screen by confirming the above-mentioned adhesion-related virulence factors that play a role in *E. faecium* pathogenesis. Although most single mutations (except for *∆fms15*) lacked a clear phenotype, this is likely due to genetic redundancy. Combining multiple mutations in a common pathway reduced pathogenesis. For example, combining mutations in *ebpABC_fm_* (or *empABC* or *fms1-5-9*) and *acm-fms11-scm* of *E. faecium* considerably limited lethality. These surface proteins are enriched in clinical isolates and aid in attachment to extracellular matrix proteins (Sillanpää et al., 2008; Hendrickx et al., 2008; Sillanpää et al., 2009; Sillanpää et al., 2010; Qin et al., 2012; Montealegre et al., 2016). The loss of *ebpABCfm* significantly attenuated biofilm formation and reduced virulence in a murine UTI model (Montealegre et al., 2016). This form of redundancy suggests that it will be especially critical to target regulatory mechanisms in *E. faecium* when developing future therapies, so that this redundancy does not compromise treatment effect.

## Data Availability Statement

The raw data supporting the conclusions of this article will be made available by the authors, without undue reservation.

## Ethics Statement

The animal study was reviewed and approved by Animal Welfare committee, University of Texas Health Science Center at Houston.

## Author Contributions

AR performed a majority of the experiments. ET performed some of the experiments. AR and ET wrote the first draft of the manuscript. BM and KS contributed to conception of the study and provided *E. faecium* isolates and mouse data. KS participated in manuscript writing. BH performed sequencing experiments. NK performed overall design of the study, contributed to data analysis and manuscript writing and editing. All authors contributed to the article and approved the submitted version.

## Funding

This work was supported by a John S. Dunn Foundation Award to NK and BM and NIH NIGMS Award R35GM129294 to NK. Funders had no role in study design, data collection or analysis, decision to publish or preparation of the manuscript.

## Conflict of Interest

The authors declare that the research was conducted in the absence of any commercial or financial relationships that could be construed as a potential conflict of interest.
