# Supplementary material for: Development and Characterization of High-Throughput Caenorhabditis elegans – Enterococcus faecium Infection Model
Source: Front Cell Infect Microbiol. 2021 Apr 29;11:667327. doi: 10.3389/fcimb.2021.667327 (PMC8116795; doi:10.3389/fcimb.2021.667327)
Supplement: Supplementary file 1 [file DataSheet_1.pdf]

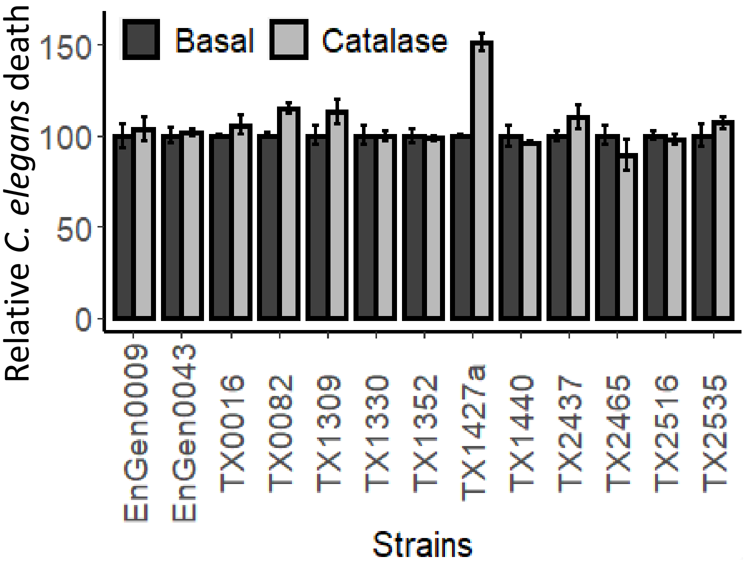

Figure S1. *E. faecium* virulence in *Enterococcus* liquid killing assay does not depend upon peroxide production. Quantification of *C. elegans* deaths upon exposure to 13 different *E. faecium* strains in the presence or absence of catalase. For each strain, killing in the absence of catalase was normalized to 100%. Three biological replicates with ~400 worms/replicate were analyzed. Error bars represent SEM.

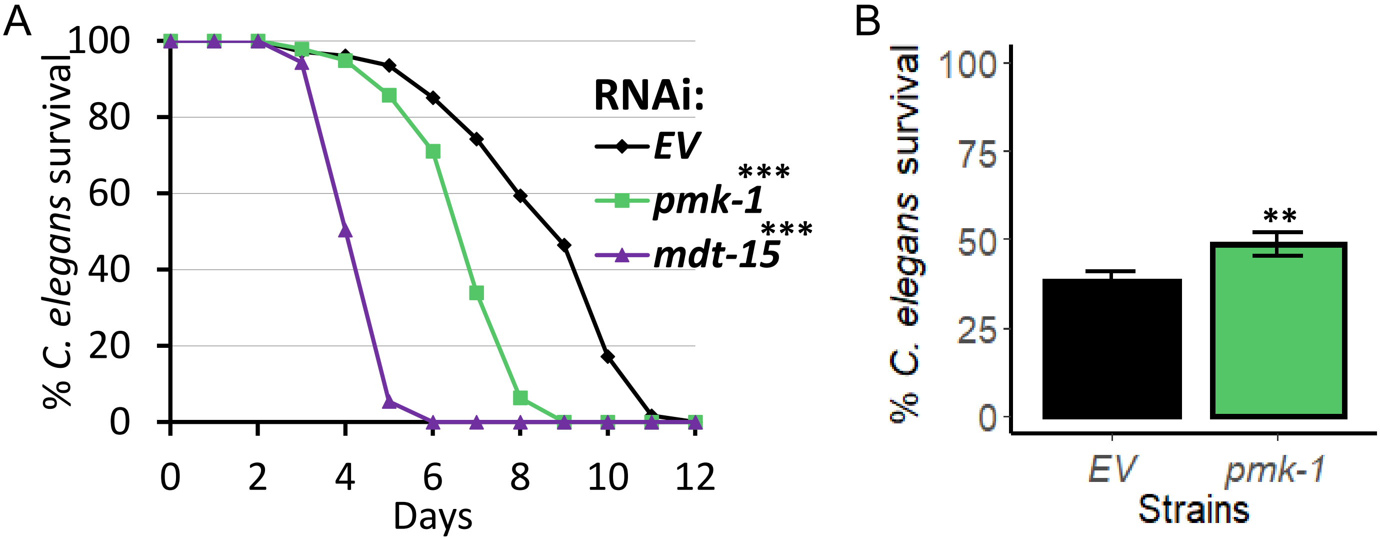

Figure S2. PMK-1 and MDT-15 are required for survival in the *Enterococcus* agar killing assay. (A) Survival curve of *glp-4(bn2)* worms exposed to *Enterococcus faecium* E007 in agar-based assay. Worms were reared on *E. coli* expressing empty vector (*EV*) as control or *mdt-15(RNAi)* or *pmk-1(RNAi)*. **(B)** Quantification of wild type or *pmk-1(km25)* worms' survival upon exposure to *E. faecium* E007 in liquid. Three biological replicates with ~60 worms/replicate for agar-based assay or ~400 worms/replicate for liquid-based assay were analyzed. Error bars represent SEM. *p* values were determined from **(A)** log-rank test and **(B)** Student's *t*-test. ** $p < 0.01$, *** $p < 0.001$.

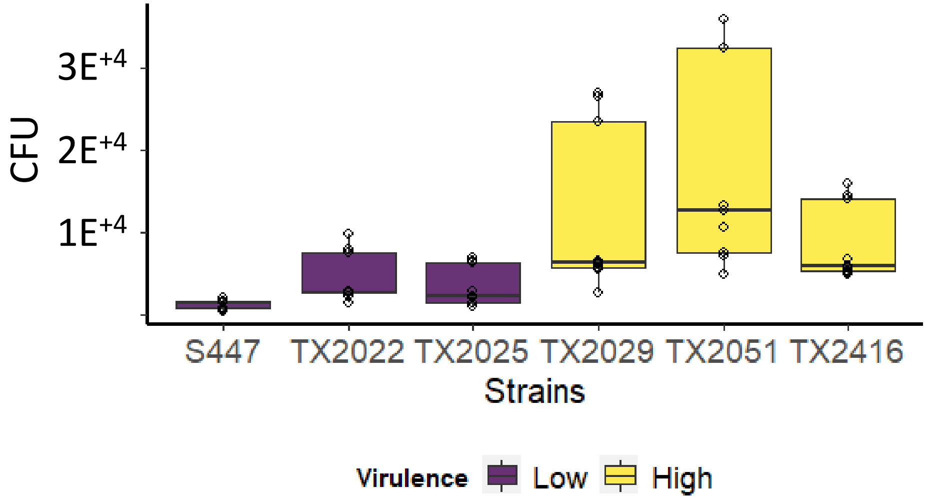

Figure S3. Highly virulent *E. faecium* strains showed higher colonization, compared to low virulence strains, in the *Enterococcus* liquid killing assay. CFU counts from *C. elegans* homogenates upon exposure to six *E. faecium* strains (three with the highest virulence in the liquid-based assay and three with the lowest virulence) in liquid-based assay. Three biological replicates with ~75 worms/replicate were analyzed.

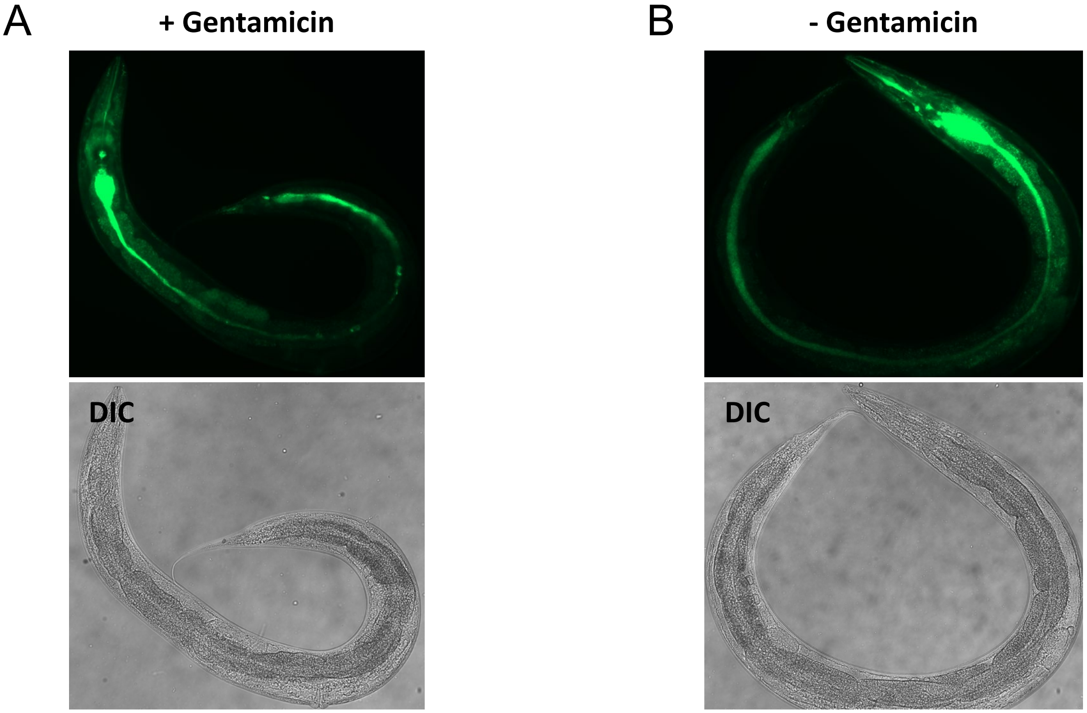

Figure S4. *E. faecium* did not attach to worms' cuticle. (A-B) Fluorescence and DIC images of *C. elegans* upon ~48 h exposure to *E. faecium* E007 in the liquid-based assay. In **(A)**, worms were treated with 20 µg/mL of gentamicin for 1 h to remove bacteria present outside the alimentary track prior to proceeding with staining or lysing. Worms were stained with acridine orange that emits green fluorescence when binds to DNA. Three biological replicates with ~30 worms/replicate were imaged.

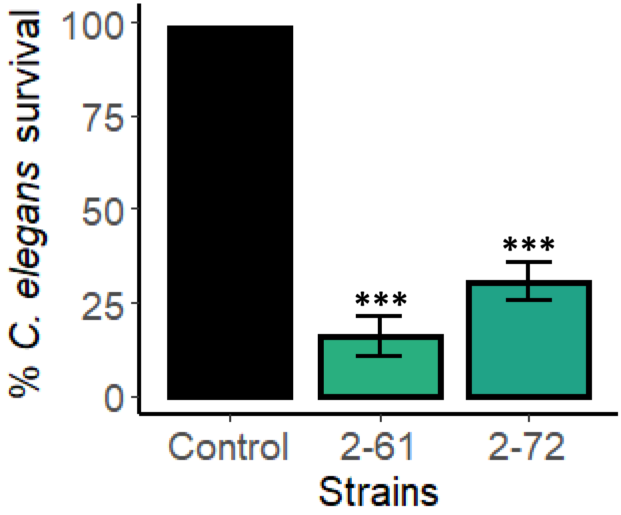

Figure S5. Filtrate collected from highly virulent *Pseudomonas aeruginosa* isolates compromised worms' survival. Survival of *C. elegans* upon 100 h exposure to filtrates from two different *Pseudomonas aeruginosa* isolates. Three biological replicates with ~200 worms/replicate were analyzed. *** $p < 0.001$.

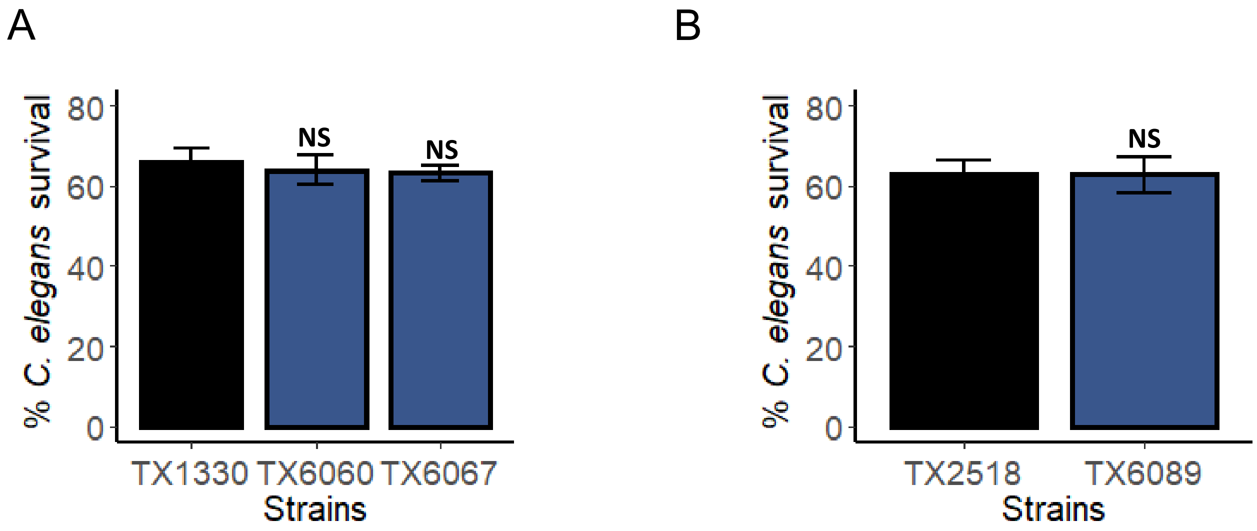

Figure S6. Several virulence factors do not play a role in the *C. elegans* – *E. faecium* liquid killing assay. Pathogenicity of several strains harboring mutations in **(A)** *gls33* and *gls20*, **(B)** *fms21-20*, were assayed in the *C. elegans* – *E. faecium* liquid-based pathosystem. Three biological replicates with ~400 worms/replicate were analyzed. Error bars represent SEM. *p* values were determined from Student's *t*-test. **NS** not significant.
